# Multiple trauma in pregnant women: injury assessment, fetal radiation exposure and mortality. A multicentre observational study

**DOI:** 10.1186/s13049-023-01084-y

**Published:** 2023-05-02

**Authors:** Paer-Selim Abback, Alison Benchetrit, Nathalie Delhaye, Jean-Luc Daire, Arthur James, Arthur Neuschwander, Mathieu Boutonnet, Fabrice Cook, Hélène Vinour, Jean-Luc Hanouz, Jean Cotte, Bruno Pastene, Viridiana Jouffroy, Tobias Gauss, Traumabase Group

**Affiliations:** 1 Department of Anesthesiology and Intensive Care, AP-HP.Nord, Beaujon Hospital, DMU PARABOL, 100 boulevard du General Leclerc, Clichy, 92110 France; 2 Department of Anesthesiology, Burn and Critical Care, Saint-Louis-Lariboisiere University Hospital, APHP, Paris, France; 3 Department of Anesthesiology and Critical Care Medicine, Hôpital Européen Georges Pompidou, AP-HP, Paris, France; 4 Radiology department, AP-HP.Nord, Beaujon Hospital, Clichy, France; 5 DMU DREAM, Department of Anesthesiology and critical care, Sorbonne University, AP-HP, Pitié-Salpêtrière Hospital, GRC 29, Paris, France; 6 Department of Anesthesiology and Intensive Care, Percy Military Teaching Hospital, Clamart, France; 7 Service d’Anesthésie et des Réanimations chirurgicales, Hôpitaux Universitaires Henri Mondor, Assistance Publique - Hôpitaux de Paris (APHP), Créteil, France; 8 Department of Anesthesiology and Critical Care, Toulouse University Hospital, University, Toulouse, France; 9 Department of Anesthesiology and Intensive Care, University Hospital of Caen, Caen, France; 10 Intensive Care Unit, HIA Sainte Anne, Military Teaching Hospital, Toulon, France; 11 Department of Anesthesiology and Critical Care, Nord Hospital, Assistance Publique Hôpitaux Universitaires de Marseille, Aix Marseille Université, Marseille, France; 12 Department of Anesthesiology and Intensive Care, AP-HP, Kremlin-Bicêtre Hospital, Kremlin-Bicêtre, France

**Keywords:** Severe trauma, Pregnancy, Initial assessment after injury, Whole-body computed tomography

## Abstract

**Background:**

Fetal radiation exposure in pregnant women with trauma is a concern. The purpose of this study was to evaluate fetal radiation exposure with regard to the type of injury assessment performed.

**Methods:**

It is a multicentre observational study. The cohort study included all pregnant women suspected of severe traumatic injury in the participating centres of a national trauma research network. The primary outcome was the cumulative radiation dose (mGy) received by the fetus with respect to the type of injury assessment initiated by the physician in charge of the pregnant patient. Secondary outcomes were maternal and fetal morbi-mortality, the incidence of haemorrhagic shock and the physicians’ imaging assessment with consideration of their medical specialty.

**Results:**

Fifty-four pregnant women were admitted for potential major trauma between September 2011 and December 2019 in the 21 participating centres. The median gestational age was 22 weeks [12–30]. 78% of women (n = 42) underwent WBCT. The remaining patients underwent radiographs, ultrasound or selective CT scans based on clinical examination. The median fetal radiation doses were 38 mGy [23–63] and 0 mGy [0–1]. Maternal mortality *(6%)* was lower than fetal mortality *(17%).* Two women (out of 3 maternal deaths) and 7 fetuses (out of 9 fetal deaths) died within the first 24 h following trauma.

**Conclusions:**

Immediate WBCT for initial injury assessment in pregnant women with trauma was associated with a fetal radiation dose below the 100 mGy threshold. Among the selected population with either a stable status with a moderate and nonthreatening injury pattern or isolated penetrating trauma, a selective strategy seemed safe *in experienced centres*.

**Supplementary Information:**

The online version contains supplementary material available at 10.1186/s13049-023-01084-y.

## Background

Severe traumatic injuries are a major cause of mortality, morbidity and handicap worldwide, [1]. The majority of deaths occur within the first 24 h following trauma. Admission to a designated trauma centre is associated with a survival benefit, [2]. As a standard of care, the initial injury assessment strategy involves standard X-rays, bedside E-FAST (Extended - Focused Assessment with Sonography for Trauma) and whole-body computed tomography (WBCT). Although this method is efficient, it is time-consuming, requires transfer to the radiology department, may reveal incidental findings unrelated to the current trauma, and carries the risk of radiation exposure, [3].

Radiation exposure is of particular concern in pregnant women since trauma is the leading cause of non-obstetric death, [4]. One out of twelve pregnant women experiences trauma during her pregnancy, [4]. Most of these injuries are mild and do not necessitate complex management. However, after motor vehicle accidents (MVAs), falls or domestic violence, the situation may require a work-up in a trauma centre. Trauma during pregnancy can result in miscarriage, preterm labour, fetal growth restriction and in utero fetal death, [5].

Fetal exposure to ionizing radiation remains a serious concern in the assessment of pregnant trauma patients. On the one hand, systematic WBCT with injection of contrast media can be considered the safest strategy to ensure that all important injuries are found; on the other hand, selective CT guided by clinical examination could be the most appropriate way to ensure minimal exposure of the fetus to radiation while providing a sufficient diagnostic evaluation of the mother. Recent evidence suggests that a cumulative radiation dose of less than 100 mGy carries very few risks at any term of pregnancy, [6] [7]. Furthermore, after initial management, some patients will require additional imaging, for example repeat computed tomography (CT) of the head or perioperative x-rays.

In this context, the present study aimed to explore whether knowledge about pregnancy in trauma is insufficient even among trauma consultants and to determine the cumulative fetal radiation dose received during the initial assessment in a cohort of pregnant women with trauma. The study group expected the knowledge level to be insufficient and that regardless of which injury assessment strategy was adopted, the radiation dose would remain beneath the 100 mGy threshold.

## Methods

### Study design and patients

This multicentre, observational, retrospective study was conducted within the TRAUMABASE network. The TRAUMABASE network manages a prospective trauma registry with 21 participating centres across France. All consecutive pregnant trauma patients admitted to one of the participating centres were included in the study. All centres perform a routine pregnancy test on admission to female patients of reproductive age. Clinical and epidemiological data were extracted from the registry or retrieved from the patient’s file: age, body mass index, gestational age (GA), medical history, length of intensive care unit (ICU) and hospital stay, mechanism and severity of trauma, prehospital and resuscitation parameters, biological and radiological data, blood product requirement, Simplified Acute Physiology Score II (SAPS II), Injury Severity Score (ISS), Sequential Organ Failure Assessment score at 24 h (SOFA score), imaging modalities for injury assessment, in-hospital mortality and maternal and fetal outcome.

### Online survey

To assess the potential knowledge gap with regard to the management of pregnant trauma patients and the risk benefits of radiation exposure, and to characterize existing practice patterns and protocols, a sample of radiology, obstetric, critical care and emergency physicians in various level-1 trauma centres in France received an online survey (Google Docs, see supplementary material 2). The questionnaire explored the initial injury assessment of pregnant patients and how confident participants felt in this particular context. All participants were urged to forward the survey questionnaire to their colleagues to increase participation (snowballing). All participants were summoned twice to respond and forward the questionnaire.

### Radiation dose calculation

No predetermined imaging or WBCT protocol was applied by any participating centre. The team in charge decided upon the diagnostic strategy and the performed WBCT protocol. A senior radiophysicist (JLD) retrospectively calculated the cumulative radiation dose received by the fetus during the initial radiological injury assessment. Only the radiation dose generated by the initial WBCT or other selective imaging on admission was considered for the study. Total irradiation exposure during the entire hospital stay was not calculated. The variables used to calculate the uterine absorbed radiation dose after CT examinations are CT scan manufacturer and model; explored organs/regions; number, length and positioning of scan series (CT protocol); for each series - tube voltage (kVp), tube current (mAs), pitch, slice thickness, volume computed tomography dose index (CTDIvol) and the dose-length product (DLP); number of detectors and collimation.

The radiation dose was calculated according to these criteria and the recommendations of the French National Institute for Radioprotection and Nuclear Safety (IRSN), [8, 9]. The CTDIvol is routinely recorded in a CT dose report. For the exams before 2015, the CTDIvol value was not available. We then used CTexpo software (SASCRAD society: Scientific and application-oriented studies and consulting in Radiology; http://www.sascrad.com) to generate an estimation of CTDIvol in these cases. Once the CTDI was known, VirtualDose software (Virtual Phantoms Inc., http://www.virtual-dose.com) was used to estimate the cumulative fetal radiation dose varying by GA with three distinctive anthropomorphic phantoms (3, 6 and 9 months of pregnancy). Monte Carlo simulations allowed us to estimate the dose received by each organ, [10]. The result was considered “Fetus total” (see supplementary material 1).

### Outcomes

The primary endpoint was the cumulative radiation dose (mGy) received by the fetus depending on the imaging strategy during the initial injury appraisal, including WBCT, CT and/or X-rays needed.

Secondary endpoints were (a) the proportion of patients receiving 100 mGy or more, (b) fetal and maternal mortality, (c) the incidence of haemorrhagic shock after trauma, defined as transfusion of 4 units of red blood cells or more within the first six hours of admission, (d) the description of the initial imaging assessment and (e) the level of agreement between participants to the survey with regard to echographic and radiological injury work-up and their medical specialty.

### Statistical analysis

Continuous data are presented as the median and interquartile range (IQR) or mean (Standard deviation) for the Trauma Injury Severity Score TRISS. Categorical data are presented as absolute values and percentages, with 95% Confidence Intervals [CI] for observed and reported rates.

### Regulatory aspects and data protection and monitoring

This registry obtained approval from the Institutional Review Board (Comité de Protection des Personnes, Paris VI and Clermont-Ferrand), and the study was specifically approved by the Review Board of the French Society of Anesthesiology and Intensive Care (SFAR; reference number IRB 00010254 − 2020–015). The trauma registry was approved by the Advisory Committee for Information Processing in Health Research (Comite Consultatif Pour le Traitement de l’information en matière de recherche dans le domaine de la santé CCTIRS, 11.305bis) and from the National Data Protection Agency (Commission Nationale de l’Informatique et des Libertés CNIL, 911,461), waiving the need for informed consent. All research was performed in accordance with relevant guidelines and regulations. The registry deploys numerous internal algorithms for data consistency and coherence; professional data monitoring is performed by trained statisticians from the Biostatistics Laboratory of Paris 7 University.

## Results

### Online survey

One hundred and twenty-four physicians responded to the survey. Most of the respondents were senior physicians (n = 79, 64%), 36 (29%) were residents and 9 (7%) were interns. Over 50% of them had more than 5 years of experience caring for multiple trauma victims, and *47%* declared having already previously taken care of a pregnant patient in a trauma context. When asked about their strategy regarding imaging assessment, *85% [79; 91]* would perform E-FAST, *65% [57; 73]* would perform WBCT and *37% [38; 45] would* perform targeted imaging based on clinical examination. The strategy regarding the specialty of clinicians is presented in Table 1. Only *31% [23; 39]* of the participants were aware of the increasing probability of harm to the fetus above a 100 mGy threshold.

**Table 1:** Initial injury assessment according to the medical specialty

Specialty	Number of responders	Targeted imaging strategy	WBCT
Anesthesiology/Critical care	46	14 *(30% [17; 43] )*	*32 (70% [57; 83])*
Obstetric/ Gynaecology	24	11 *(46% [26; 67])*	13 *(54% [34; 74])*
Radiology	23	12 *(52% [32; 72])*	11 *(48%[28; 68])*
Emergency medicine	31	9 (29% *[13; 45])*	22 (71% *[55; 87])*
*All disciplines*	*124*	*46 (37% [28; 45])*	*78 (63% [54; 71])*

Data are presented as n(%) and 95% Confidence Intervals in the specialty or among all responders.

### Cohort study

**Fig. 1:**
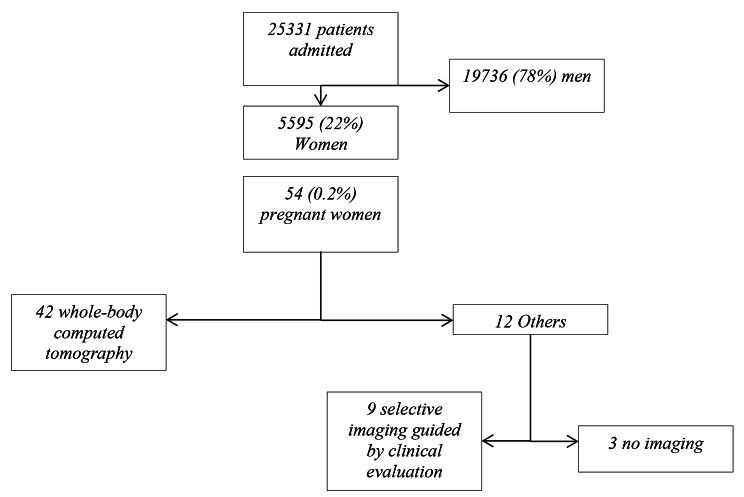
Flow Chart of patients included in the study

From September 2011 until December 2019, 25 331 patients were admitted to one of the participating centres for major trauma. Among these, 5595 *(22%)* were women, and 3497 *(62%)* were 50 years old or younger. Fifty-four (0.2%) of all patients, representing 1.5% of women aged 50 or less, were identified as pregnant in the registry, and 9 of them *(17%)* were unknown and diagnosed during routine pregnancy testing on admission *(*Fig. 1*).*

Patient characteristics at admission are reported in Table 2. The main mechanism of injury was MVA (n = 37, 69%) of moderate to high velocity. Five patients (9%) presented with penetrating injury: 4 patients with multiple stab wounds and 1 patient with a gunshot wound to the head after suicide attempt. Ten patients (19%) had a Glasgow coma scale (GCS) less than or equal to 13 on scene, and 12 patients (22%) were intubated on scene. No patient was intubated in the resuscitation room. Twenty out of 54 (37%) presented with a severe traumatic load (ISS > 15). The median GA was 22 weeks [12–30]. A total of 35% were in their first trimester, 33% were in their second trimester, and 29% were in their third trimester. The term of pregnancy was unknown for 2 women.

### Primary outcome

In 42 of 54 cases *(78% [67; 89])*, the initial injury appraisal included a WBCT, in 9 of 54 *(17% [7; 27])* appraisal consisted of selective imaging guided by clinical evaluation and in 3 of 54 *(6% [0; 12])* cases no imaging was performed *(*Fig. 1*).* For 7 of 42 patients, imaging files were not retrieved on the server, and in 6 of 42 cases, it was impossible to obtain access to the image files. After exclusion of 16 of 54 cases without image files (13 of 16 were unavailable, 3 of 16 had no imaging), 29 cases with WBCT and nine selective injury assessment cases were analysed. The median fetal radiation dose received by all patients was 23 mGy [0.5–43]. When WBCT was performed, the median fetal radiation dose was 38 mGy [23–63] compared to 0 mGy [0–1] when a clinically guided CT examination was adopted (Table 3).

Among the 29 women assessed by WBCT, two fetuses received a radiation dose above 100 mGy (110.89 mGy and 112.99 mGy, respectively). One of them (GA 18 weeks) was exposed to a radiation dose greater than 100 mGy in the context of repeated abdominal and pelvis imaging with and without arterial and venous phase contrast enhancement. For the second, fetal death was diagnosed before WBCT; concordantly, a higher dose was tolerated in the trauma appraisal of the mother.

**Table 2:** Patient’s characteristics

**Age, y**	29 [26–34]
**BMI (kg.m** ^**− 2**^ **)**	25.3 [23.3–27 ;3]
**Median gestational age (weeks)**	22 [12–30]
**Mechanism of trauma**	
- MVA	37 (69%)
- Gunshot wounds / Stab wounds	5 (9%)
- Falls	12 (22%)
**Severity Scores**	
- SAPS 2	14 [8–21]
- ISS	9 [2–20]
- ISS > 15	20 (37%)
- SOFA at 24 h	0 [0–3]
***Maternal mortality***	*3 (6%)*
**Predicted maternal mortality**	
- *TRISS (mean(SD))*:	*8% (12%)*
**Clinical data at first medical evaluation**	
- Cardiac arrest	2
- GCS	15 [14–15]
- Systolic blood pressure < 90 mmHg	11 *(20%)*
- Heart rate > 120/min	7 (13%)
- Vasopressors (adrenaline, noradrenaline)	8 (15%)
- Out-of-hospital intubation	12 (22%)
**Clinical data upon arrival at the hospital**	
- Cardiac arrest	1
- Systolic blood pressure < 90 mmHg	4 *(7%)*
- Heart rate > 120/min	5 (9%)
- Vasopressors (adrenaline, noradrenaline)	8 (15%)
- In-hospital intubation	0
**Biological data upon arrival at the hospital**	
- lactate (mmol/L)	1.4 [1-1.9]
- base excess (mmol/L)	-4.9 [-6.4—3.5]
**Transfusion (RBC)**	
- pre-hospital	2
- in-hospital	12 (22%)
**Surgery in the first 24 h**	22 (41%)
- orthopedic surgery	12
- exploratory laparotomy	5
- maxillofacial surgery	2
- emergency cesarean section	2
- neurosurgery	1
**ICU length of stay, d**	2 [1–6]
**Hospital length of stay, d**	6 [2–17]

Data are presented as medians [IQR 25-75, absolute value (percentage %) or mean (standard deviation) for the TRISS.

BMI Body Mass Index, MVA motor Vehicle Accident, SAPS II Simplified Acute Physiology Score II, ISS Injury Severity Score, SOFA Sequential Organ Failure Assessment, GCS Glasgow Coma Score, ICU Intensive Care Unit, RBC Red Blood Cells, TRISS Trauma Injury Severity Score.

Nine patients had a selective imaging strategy. In 3 of 54, the fetus was in the irradiation field, but the clinical assessment alone limited the injury appraisal to a selective strategy with regional CT and/or standard radiographs. This reduced the fetal radiation dose in one patient with multiple abdominal stab wounds to 51 mGy after a trunk-only scan. One patient had a standard pelvic X-ray, which was sufficient to rule out a pelvic fracture (fetal radiation dose < 1 mGy); one patient required two perioperative lumbar radiographs in the operating room after spine surgery (fetal radiation dose < 1 mGy). The initial assessment of this patient consisted of magnetic resonance imaging (MRI). For the 6 remaining patients, the fetus was not exposed to any radiation because it was out of the radiation field.

**Table 3:** Cumulated radiation dose (mGy) received by the fetus according to the type of injury assessment method and the gestational age

	WBCT (n = 29)	Imaging guided by clinical evaluation (n = 9)
**Median gestational age (weeks)**	23 [12–30]	14 [14–20]
**Fetal radiation dose (mGy)**	38 [23–63]	0 [0–1]

Data are presented as medians [IQR 25-75]; WBCT Whole Body Computed Tomography, mGy milliGray

### Secondary outcomes

#### Maternal mortality

Three pregnant women died as a consequence of their trauma (6%). One woman died on day one after multiple stab wounds to the trunk and initially resuscitated haemorrhagic cardiac arrest on scene. Two other patients succumbed after MVAs at 24 and 30 weeks gestation. One patient was in cardiac arrest on scene and died within a few hours of arrival due to uncontrolled haemorrhagic shock despite resuscitation and damage control laparotomy. The other patient suffered massive craniofacial trauma with severe intracranial hypertension due to brain oedema despite decompressive craniectomy. An emergency caesarean section was performed because of fetal bradycardia.

#### Fetal mortality

An obstetric ultrasound was at admission performed to assess fetal vitality in 39 *of* 54 patients (72%), in the resuscitation room (90%) or in the first 24 h (10%). Five patients were considered too premature in their pregnancy to perform this exam. Nine of 54 fetuses (17%) died: one uterine rupture at 10 weeks, one early traumatic miscarriage following a high velocity MVA, six in utero fetal deaths and one fetus at 38 weeks requiring emergency caesarean section due to extreme bradycardia (maternal haemorrhagic shock) but was stillborn. Most of them (7 of 9) died upon arrival in the trauma bay as diagnosed by fetal ultrasound.

Two patients underwent therapeutic abortions after the discovery of polymalformative syndromes. The first was diagnosed in the context of trauma, and the second was diagnosed later in pregnancy following fetal irradiation of less than 1 mGy resulting from a clinically guided initial assessment.

#### Maternal haemorrhagic shock

Six patients presented with haemorrhagic shock. Four patients required immediate surgery before WBCT. The two remaining patients underwent WBCT first after haemodynamic stabilization. In total, five patients required emergency surgeries: three splenic injuries, four hepatic injuries, one uterine rupture, and two wounds of the digestive tract (caecum and small intestine). One patient needed embolization of the two hypogastric arteries due to active haemorrhage complicating a pelvic fracture after an emergent caesarean section for fetal bradycardia on admission. Among those six patients, one died, while five fetuses did not survive.

#### Imaging injury assessment

Among the 48 (89% of all patients) E-FAST exams performed upon arrival in the resuscitation room, 8 (16%) were positive: seven cases of haemoperitoneum, two of haemothorax and one of pneumothorax.

A WBCT was ordered for 42 of 54 patients (78%): 35 were performed immediately after the first clinical assessment in the resuscitation room, four after fetal extraction (two emergency caesarean sections with only one child alive and two intraoperative fetal extractions for deceased fetuses) and four after emergency surgery (exploratory laparotomy).

Nine patients (17%) had a clinically guided imaging strategy. These were haemodynamically stable, with a negative E-FAST and no clinical signs of severe injury. A patient who presented with a sensory-motor deficit of the lower limbs underwent lumbar spine MRI demonstrating a T12 burst fracture with spinal cord compression. Three had CT guided with clinical examination (head after gunshot, trunk after multiple stab wounds, head and spine after loss of consciousness and pain). Two patients had X-rays focused on painful areas (limbs, thorax). Three patients did not have any imaging other than E-FAST.

*Among the 54 included patients, 9 of them were unknown and diagnosed during routine pregnancy testing on admission. Seven of them had a WBCT while 2 had clinically guided strategy. On the opposite, 7 fetuses died upon arrival in the trauma bay as diagnosed by fetal ultrasound, 6 of them having a WBCT. So if we try to calculate a WBCT rate where a viable pregnancy was known at the time of decision, this would be 29 WBCT with viable fetuses among 38 patients, so a WBCT rate of 76%.*

## Discussion

This study explored the knowledge level regarding pregnancy and trauma, and assessed maternal and fetal radiation exposure [11]. First, there seems to be a considerable knowledge gap among providers treating these cases. Second, fetal radiation exposure was considerably higher with systematic use of WBCT for injury assessment compared to a targeted image protocol based on clinical suspicion alone. However, in the vast majority of cases, the cumulative fetal radiation exposure remained below a potentially harmful 100 mGy threshold. Fetal mortality in our cohort was higher than maternal mortality: *17%* (9 fetuses) and *6%* (3 women), respectively.

A recent French multicentre prospective study, [12] followed a cohort of 319 pregnant women exposed to an abdominopelvic scan for various diagnostic purposes and compared these to a matched nonexposed cohort. In the exposed cohort, three hundred and twelve fetuses (97.5%) received a radiation dose below 50 mGy and 99.4% below 100 mGy. There was no significant difference between the two groups regarding miscarriage, in utero fetal death, fetal growth restriction or malformations (7.8% vs. 7.2%; p = 0.88). These data suggest that a fetal radiation dose of less than 100mGy is safe.

The place of WBCT as part of a routine trauma work-up remains a matter of debate. Several large retrospective studies with adequate confounder control and adjustment for severity demonstrate a reduction in mortality with initial and routine use of WBCT, [13–15]. A multicentre randomized controlled trial did not find any decrease in mortality when performing WBCT versus a clinically guided CT examination (16% vs. 16%, p = 0.92) in patients admitted for suspicion of severe traumatic injuries, [16]. Furthermore, radiation doses were significantly higher in the systematic WBCT group than in patients assessed with a clinically oriented CT scan. (20.9 mSv [20.6–20.9] vs. 20.6 mSv [9.9–22.1]; p < 0.0001). However, despite being significant, this difference is probably not clinically relevant, particularly considering a high crossover rate between both groups. Other authors contend that WBCT detects lesions unrelated to the current trauma, adding anxiety for the patient and increasing medical costs (longer hospital stay, complementary examinations), [17]. However, as Salim demonstrated in a prospective trial, systematic use of a WBCT in blunt trauma unveils 20% of injuries otherwise undetected, [18]. This finding is important in particular in specific patient groups, such as pregnant women, which are difficult to examine and assess. The difficulties associated with pregnancy-related anatomical and physiological changes can make the clinical examination unreliable and a WBCT indispensable.

Trauma E-FAST provides crucial information for prompt decision-making and even more so in pregnant patients: (1) identification of intraabdominal free fluid for unstable patients and (2) early diagnosis of absence of fetal heart activity. Consequently, any injury assessment protocol should associate early E-FAST with quick obstetric ultrasound. Based on the results, several strategies can be adopted depending on the situation. In stable patients with a moderate and nonthreatening injury pattern, a clinically guided imaging strategy associating E-FAST, X-ray or selective CT, and lower irradiation can be applied in experienced centres. In the present cohort, no complications or missed injuries were observed. This selective imaging strategy could also be sufficient in unstable patients with isolated penetrating trauma. However, the results also demonstrate that in most cases, even with a strategy including a WBCT, the final radiation dose remains below a potentially harmful threshold of 100 mGy.

The results and the survey underscore considerable discrepancies in imaging protocols to assess pregnant trauma patients across different centres resulting in various levels of fetal radiation exposure. For example, in one hospital, 6 of 14 patients were exposed to radiation during portal acquisition, with exposure equivalent to three abdominal irradiations, limited to two in all other centres. In fact, survey results illustrate the knowledge gap with regard to fetal irradiation and exposure and exemplify the discrepancies among different specialties. Radiologists tend to have a higher threshold to perform a WBCT than practitioners in anaesthesia/critical care.

These observations of the cohort study combined with the survey results plead the case for standardized institutional imaging protocols for injury assessment in pregnant patients. The objective of such a protocol is to associate a high level of sensitivity to detect maternal injuries while keeping fetal irradiation as low as possible. As demonstrated by this cohort, the incidence of pregnant major trauma patients was low. This contributes to uncertainty and possibly disagreement between the involved clinicians and heterogeneous practice patterns. Institutional protocols help to reduce uncertainty, conflict and discrepancy. In the development of this imaging protocol, it is important to keep in mind that, as the Royal College of Radiologists describes, “…*The health of the mother takes precedence over the health of the fetus and, if appropriate, modification of pathways should be decided by the trauma team leader and consultant radiologist.”*, [19].

### Limitations

This study is characterized by a few limitations. The cohort appears small but reflects recruitment from a large sample of designated trauma centres across France based on routine and systematic screening. The incidence of pregnant patients admitted for trauma seems indeed low [5]. Due to the retrospective design, this study was not able to determine whether GA influenced the initial strategy. However, we can suppose that not only GA but also fetal status (alive or not) on admission played a role in the assessment strategy. Furthermore, for a few patients admitted prior to 2015, it proved impossible to retrieve detailed imaging reports.

It would be preferable to examine the medium- and long-term evolution of exposed fetuses. Theoretically selective imaging carries the risk of missed injuries, but all patients were observed over a few days in the hospital. Any clinically pertinent injury would have been identified; with regard to the low risk associated with a selective strategy in selected cases, selection bias cannot be excluded. Given the small sample size, the risk of lack of power in detecting infrequent complications is real.

We did not study the possible radiation of secondary exams such as control CT. Without the time constraint of the initial urgent assessment, the relatively nonurgent aspect of these exams allows the adoption of diagnostic strategies to reduce fetal exposure to a minimum.

We decided to estimate the absorbed dose in mGy determined by Monte Carlo simulations and not the effective dose in mSv. To our knowledge, there are unfortunately no official methods to assess the effective dose (mSv). All recommendations about the clinical critical thresholds were made in mGy.

## Conclusion

This multicentre observational study confirms that pregnant women are rarely victims of severe trauma, but the associated morbidity and mortality are substantial. It seems that performing total-body CT scanning immediately on admission is not associated with fetal radiation doses above the recommended threshold of 100 mGy. Furthermore, in experienced centres, among the selected population with either a stable status with a moderate and nonthreatening injury pattern or isolated penetrating trauma, a selective strategy seemed safe. There is a knowledge gap even among experienced clinicians and a need for institutional initial injury assessment protocols.

## Electronic supplementary material

Below is the link to the electronic supplementary material.

Supplementary Material 1

Supplementary Material 2

## Data Availability

The datasets used and/or analysed during the current study are available from the corresponding author on reasonable request.
