# Supplementary material for: Multiple trauma in pregnant women: injury assessment, fetal radiation exposure and mortality. A multicentre observational study
Source: Scand J Trauma Resusc Emerg Med. 2023 May 2;31:22. doi: 10.1186/s13049-023-01084-y (PMC10152762; doi:10.1186/s13049-023-01084-y)
Supplement: Supplementary file 1 — Supplementary Material 1 [file 13049_2023_1084_MOESM1_ESM.pdf]

<u>**Supplementary material**</u>

Supplementary material 1
Screenshot of VirtualDose software displaying a 6-month pregnant woman going through a TAP-CT.

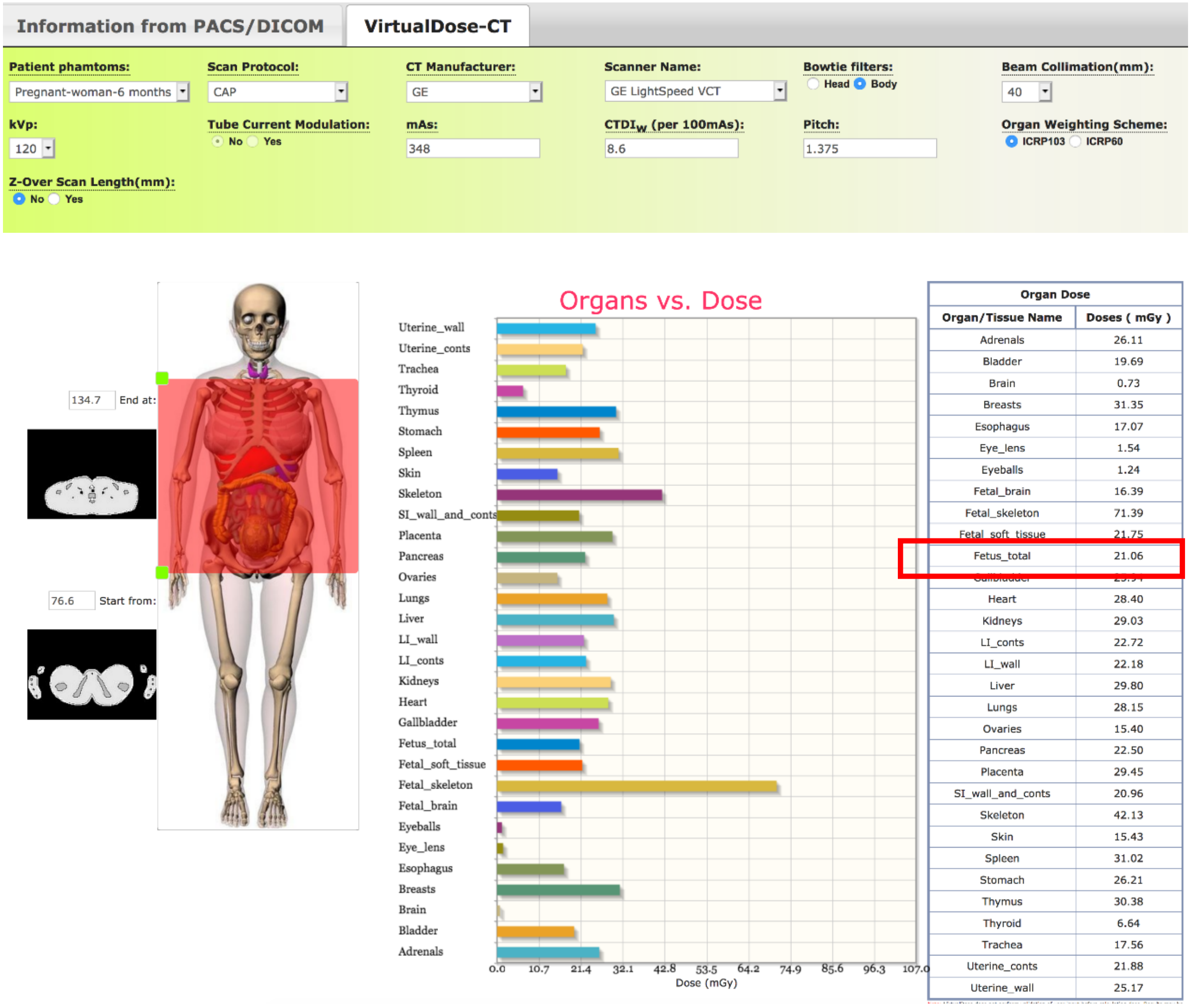

Supplementary material 2: online survey

Practice evaluation regarding pregnant women victim of severe trauma

1. E-mail address

2. What is your status ?

- intern
- resident
- senior doctor / professor

3. Which specialty are you practicing ?

- Critical care medicine
- Emergency medicine
- Obstetrics
- Radiology

4. Where do you work ?

5. How old are you ?

- 20-25 yo
- 26-30 yo
- 31-35 yo
- 36-40 yo
- 41-45 yo
- 46-50 yo
- 51-55 yo
- 56-60 yo
- 61-65 yo
- 66-70 yo
- 70-75 yo

6. What is your personal experience on taking care of multiple trauma victims ?

- less than 6 months experience
- 6 months to 2 years experience
- 2 to 5 years experience
- more than 5 years experience

7. Have you ever taken care for a pregnant woman suspected of multiple trauma?

- yes
- no

8. If so, how often ?

- 1 or 2 times a year
- 2 to 5 times a year
- more than 5 times a year

9. Is there a dedicated protocol in the hospital you work in for this specific situation ?

- yes
- no
- I don't know

10. In which facility would you take care of a pregnant woman suspected of a severe trauma ?

11. What would be your initial imaging assessment?

- Chest and pelvis radiography
- E-FAST (Extended-Focussed Assessment with Sonography for Trauma)
- Obstetrical ultrasound
- MRI
- Whole Body Computed Tomography (WBCT)
- Clinically-guided imaging assessment

12. Explain your choices

13. What are the benefits of performing E-FAST?

14. Why wouldn't you perform a WBCT in this peculiar situation?

15. If you perform a WBCT, would you use contrast injection? Why?

16. What are the potential consequences of exposing a fetus to ionizing radiations ?

17. What is the threshold (in mGy) below which ionizing radiations received by the fetus are supposed to be safe?

- 20 mGy
- 50 mGy
- 100 mGy
- 200 mGy
- 500 mGy
- I have no clue

18. Do you think interventional radiology is allowed in pregnant women? Explain.

19. If you ever had to care for a pregnant woman victim of severe trauma, how would you describe your feeling of control regarding the situation? (On a scale of 1 to 10; 1: no control of the situation; 10: full control)

20. If you ever had to be presented with the same situation, how confident would you now feel? (scale of 1 to 10)
