# Supplementary material for: Multiple trauma in pregnant women: injury assessment, fetal radiation exposure and mortality. A multicentre observational study
Source: Scand J Trauma Resusc Emerg Med. 2023 May 2;31:22. doi: 10.1186/s13049-023-01084-y (PMC10152762; doi:10.1186/s13049-023-01084-y)
Supplement: Supplementary file 2 — Supplementary Material 2 [file 13049_2023_1084_MOESM2_ESM.docx]

Dear Ladies and Gentlemen,

We would like to thank the reviewers for their thoughtful and pertinent review. Please find our replies to their comments points by point (comments in italics). Corresponding modifications have been highlighted in red and italics in the “Manuscript”.

We hope the modifications and comments will be pertinent enough, and group would be much obliged if the editorial board would reconsider our manuscript for publication.

Respectfully,

PS Abback, MD.

*Reviewer #1: Dear authors,

thank you for your work.

I find the manuscript interesting and well written, I have only some minor comments:

on page 10 line 190 wold -> would*

We thank the reviewer for this comment. Correction has been made.

*You could consider adding a flow-diagram (even if the text is already very precise regarding this aspect).*

We thank the reviewer for this comment. A flow chart has been added (Figure 1).

*You could expand your conclusions:
- In the abstract you write "Among the selected population with either a stable status with a moderate and nonthreatening injury pattern or isolated penetrating trauma, a selective strategy seemed safe", this aspect is motivated in your discussion, where you also add "-- selective CT and lower irradiation can be applied in experienced centres", I find that adding "in experienced centres" would be appropriate also in the abstract.

In the conclusion of the article you could add a resume in line with that of the abstract  "(In experienced centres) Among the selected population with either a stable status with a moderate and nonthreatening injury pattern or isolated penetrating trauma,  a selective strategy seemed safe ".*

We thank the reviewer for this comment. These precisions have been added to both conclusions.  *Reviewer #2: Fortunately, severe injuries in pregnant women is a rather seldom event.
The present paper reports about 52 women documented during a 9 year period from 21 trauma centers.
This means about one case in four years (if the centers have similar size and provided data for the whole period).
In such situations a registry analysis is an optimal source of evidence.*

We thank the reviewer for this comment.  *The authors also added the results of a survey (n=124)
Two third of the respondents would consider WBCT even in pregnant women.
Table 1 should also contain a summary line where the results of all disciplines were merged.*

We thank the reviewer for this comment. This line has been added.

*The primary outcome was prevention from radiation for the fetus, espacially in case of a whole-body CT.
What were the inclusion criteria for the registry? Any trauma admission? Lower quartile of ISS of the included women was 2.*

We thank the reviewer for this comment. The Traumabase registry prospectively collects data for all patients consecutively admitted to a participating major Trauma center for a suspicion of severe trauma according to the Vittel criteria, based on national french triage guidelines (1).

1 : Hamada SR, Gauss T, Duchateau F-X, Truchot J, Harrois A, Raux M, et al.Evaluation of the performance of French physician-staffed emergency medical service in the triage of major trauma patients. J Trauma Acute Care Surg. 2014;76:1476–83.

*The observed rate of WBCT (78%) is higher than the rate reported in the survey (about 65%).
Please provide a 95% confidence interval for the observed rate.*

We thank the reviewer for this comment. 95% Confidence Intervals were added for all reported and observed rates in the manuscript.

*Was the pregnancy status known in all 42 cases with WBCT?
In 9 women, the pregnancy status was unknown before the accident.
It was also stated that in one case the fetus was dead already when CT was performed.
Among the other non-surviving fetuses, did they die after CT scan (i.e., no influence on decistion to do CT)?*

We thank the reviewer for this comment. Due to the retrospective work, we cannot know whether the pregnancy status was known when the CT was performed. As indicated, in 7 cases, the fetus died upon arrival in the trauma bay as diagnosed by fetal ultrasound, so the CT was performed after the fetal death.

*Please, try to calculate a WBCT rate in pregnant women where the pregnancy (with a vital fetus) was known (so exclude from the denominator those cases where the fetus was initially identified to be already dead, and cases wher pregnancy was unknown at the time of decision). This rate would fit to the rate observed in the survey.*

We thank the reviewer for this comment. Among the 54 included patients, 9 of them were unknown and diagnosed during routine pregnancy testing on admission. Seven of them had a WBCT while 2 had clinically guided strategy. On the opposite, 7 fetuses died upon arrival in the trauma bay as diagnosed by fetal ultrasound, 6 of them having a WBCT. So if we try to calculate a WBCT rate where a viable pregnancy was known at the time of decision, this would be :

42 WBCT – 7 unknown pregnancy – 6 dead fetuses on admission = 29 WBCT of known viable or suspected fetuses.

54 patients – 9 unknown pregnancies – 7 early dead fetuses = 38.

So, a WBCT rate of 76%

These results have been integrated to the text.

*Minor points:

- TRISS (Table 2): although the distribution is rather skewed, the expected mortality rate based on TRISS should be presented as a mean value.*

*Only this value could reasonalby be compared to the observed mortality rate (here one decimal is OK).*

We thank the reviewer for this comment. The median expected rate based on TRISS is now presented as a mean value in the manuscript.

*- Hospital mortality should be added to Table 2*

We thank the reviewer for this comment. This line has been added.

*- Due to the small samle size (54 pregnant women), percentages should be provided as rounded intergers only (check tables and text).*

We thank the reviewer for this comment. Correction has been made for the corresponding results.

*- p 15, line 284:  in 39 of 54 patients*

We thank the reviewer for this comment. Correction has been made.  *Reviewer #3: A well-constructed study examines the outcomes of 54 pregnant females seen in multiple French trauma centers. Specific emphasis was placed on admission imaging with modalities ranging from whole-body CT exposure to selective CT use to various applications of ultrasound. Fortunately, fetal exposure averaged 38 mGy with the range of 23-63 mGy. One hundred mGy is noted as a threshold for significant fetal risk.*

We thank the reviewer for this comment.

*I have a small number of questions.
Can the authors provide any additional details of death by patient group? Despite avoidance of the mGy threshold, was there any evidence of radiation injury?*

We thank the reviewer for this comment. No evidence of radiation injury could be identified. Most of the fetal deaths occured very early, mostly because of the trauma itself or because of maternal instability. Even more, most of them where before the imaging assessment or within the first hours with no evidence of radiation injury.

Only two polymalformative syndromes were identified in the cohort. The likelihood for a causal link radiation exposure in the context of the diagnostic work up is low. The first syndrome was diagnosed in the context of the trauma, before WBCT. The second case was exposed to a low total radiation dose of less than 1 mGy; a causal link is unlikely. This information has been added to the manuscript.

*I believe the authors should acknowledge the relatively small sample size.*

We thank the reviewer for this comment. We tried to emphazise this point in the limitations section.

*The authors appropriately identify differences between disciplines in the use of radiation in patient evaluation. I ask them to make some concluding recommendations for imaging in the setting of trauma in the pregnant patient; for example, should imaging begin with ultrasound with use of CT only if necessary? Is a general curriculum needed for imaging of the pregnant trauma patient?*

We thank the reviewer for this comment. As discussed, such imaging should be standardized in each center. As for any suspected severe trauma, such protocol should be starting with E-FAST and completed with CT, excepted in a selective population in experienced centers where a selective strategy seemed safe (added In the conclusion).

*Overall, this study is well written and requires only summary statements as briefly described above and minor editing for grammar and usage.*

We thank the reviewer for this comment.
